# Multiplexing Genetic and Nucleosome Positioning Codes: A Computational Approach

**DOI:** 10.1371/journal.pone.0156905

**Published:** 2016-06-07

**Authors:** Behrouz Eslami-Mossallam, Raoul D. Schram, Marco Tompitak, John van Noort, Helmut Schiessel

**Affiliations:** 1 Institute Lorentz for Theoretical Physics, Leiden University, Niels Bohrweg 2, 2333 CA Leiden, The Netherlands; 2 Biological and Soft Matter Physics, Huygens-Kamerlingh Onnes Laboratory, Leiden University, Niels Bohrweg 2, 2333 CA Leiden, The Netherlands; 3 Department of Bionanoscience, Kavli Institute of Nanoscience, Delft University of Technology, Lorentzweg 1, 2628 CJ Delft, The Netherlands; Tel Aviv University, ISRAEL

## Abstract

Eukaryotic DNA is strongly bent inside fundamental packaging units: the nucleosomes. It is known that their positions are strongly influenced by the mechanical properties of the underlying DNA sequence. Here we discuss the possibility that these mechanical properties and the concomitant nucleosome positions are not just a side product of the given DNA sequence, e.g. that of the genes, but that a mechanical evolution of DNA molecules might have taken place. We first demonstrate the possibility of multiplexing classical and mechanical genetic information using a computational nucleosome model. In a second step we give evidence for genome-wide multiplexing in *Saccharomyces cerevisiae* and *Schizosacharomyces pombe*. This suggests that the exact positions of nucleosomes play crucial roles in chromatin function.

## Introduction

DNA molecules are much longer than the cells that contain them. This requires their compaction, which introduces also an opportunity: the regulation of transcription through a differentiated fashion of DNA packaging. In eukaryotes DNA molecules can guide their own packaging into nucleosomes by having the desired mechanical properties (stiffnesses and intrinsic curvature) written into their base-pair (bp) sequence. This has been referred to as the “nucleosome positioning code” [1] (for earlier versions of this idea see e.g. [2] and [3]). Nucleosomes are the fundamental packaging units of eukaryotic DNA, where 147 bp are wrapped in a 1 3/4 lefthanded superhelical turn around an octamer of histone proteins [4]. As the DNA is strongly deformed when wrapped around the histones, sequence-dependent geometrical and mechanical properties could—at least locally—overrule other effects that also influence nucleosome positioning like the presence of proteins that compete for the same DNA stretch or the action of chromatin remodellers [5].

In the present study we ask the question whether mechanical information could be written into DNA molecules. We focus here on the positioning of nucleosomes along eukaryotic DNA, but we stress that such information might also be found in the DNA of the other two domains of life, affecting e.g. the positions of archeal histones in Archaea [6] and that of supercoils in bacteria [7]. We ask first whether the mechanical properties of the base-pair (bp) sequence alone can explain the nucleosome positioning rules [3, 8]: high affinity sequences have on average more AA, TT and TA steps at positions where the minor groove faces inward towards the octamer and GC steps where it faces outwards (DNA molecules with a propensity for ring formation exhibit similar rules [9]). We then ask whether one can position nucleosomes freely on top of genes, i.e. whether the classical genetic and the mechanical information can be multiplexed. Multiplexing is well-known in daily life technology, allowing e.g. to carry several phone conversations on the same wire and has been speculated to occur in nucleotide sequences [10]. And finally we look for evidence that this kind of multiplexing occurs in real genomes.

To address these questions it was crucial to overcome the usual limitations that hamper this field. The main challenge is the immensity of the number of sequences, 4^147^, that can be wrapped into a nucleosome. A densely packed DNA molecule containing all these ∼10^88^ sequences would fill the volume of five Milky Ways. The genome of yeast with its 12 million bp only contains the 10^−81^th part of this gigantic space. Even experimentally starting with a much bigger pool of 5 × 10^12^ sequences, Lowary and Widom [8] only found about 30 high affinity sequences through competitive binding to histone octamers. In all these cases the problem is that one has to choose the pool of sequences upfront and only a tiny fraction of them have the desired properties. Here, we introduce a computational approach, the Mutation Monte Carlo method (MMC), that overcomes these limitations. We apply it to a coarse-grained nucleosome model that is simple enough to allow effective computations for a large number of bp sequences, but precise enough to recover the well-known positioning rules. A variant of the MMC method will allow us to demonstrate multiplexing of genetic and mechanical information and to explain its underlying principles. Finally, a bioinformatics approach will provide evidence for multiplexing on two eukaryotic genomes.

## Methods and Models

### Nucleosome model

**Fig 1:**
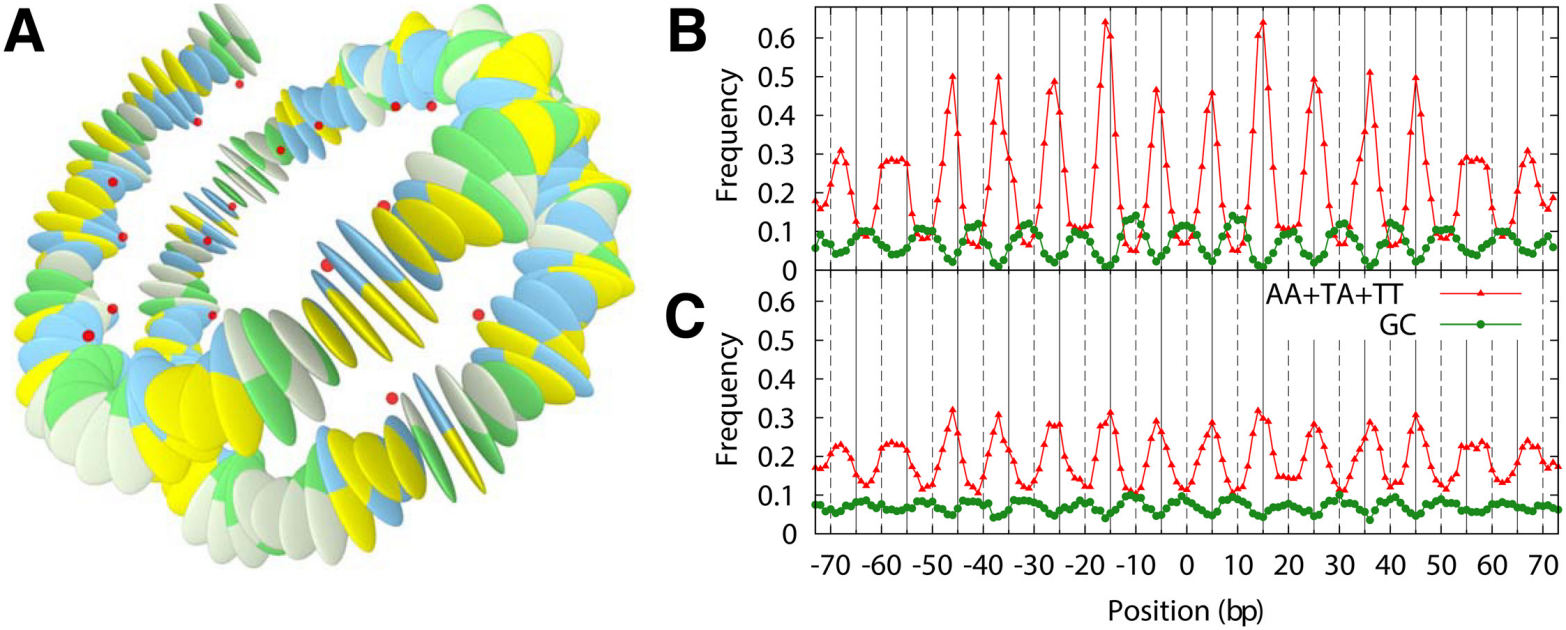
Nucleosomal DNA model with bp step dependent mechanical properties. (A) The rigid base-pair model is forced, using 28 constraints (indicated by red spheres), into a lefthanded superhelical path that mimics the DNA conformation in the nucleosome crystal structure [4]. (B) Fraction of dinucleotides GC and AA/TT/TA at each position along the nucleosome model found in 10 million high affinity sequences produced by MMC at 100 K. The solid and dashed lines indicate minor and major groove bending sites; the nucleosome dyad is at 0 bp. The model recovers the basic nucleosome positioning rules [1, 3]. (C) Same as (B), but on top of 1200 coding sequences (produced by sMMC). The same periodic signals are found albeit with a smaller amplitude.

Our nucleosome model consists of a 147-bp-long DNA molecule represented by the rigid base-pair model that is forced into a superhelical conformation through constraints that mimic the binding of 28 DNA phosphates to the protein core, see Fig 1A. We first describe the coarse-grained DNA model and then explain how we constructed the constraints.

We represent the DNA by the rigid base-pair model which describes the conformations of the DNA double helix solely by the positions and orientations of its base-pairs that are represented by rigid plates [11, 12]. This leaves six degrees of freedom per bp step, three translations—shift, slide, rise—and three rotations—twist, roll, tilt. We assume that the six degrees of freedom of a given bp step have preferred intrinsic values (dependent on its chemical composition) and that deviations from these values incur a mechanical energy cost quadratic in this deformation:

$$E_{\text{el}}=\frac{1}{2}{\left(q-q^{0}\right)}^{T}Q\left(q-q^{0}\right).$$ (1)

Here *q* is a six-component vector that contains the 6 degrees of freedom whose intrinsic values are given by *q*^0^ and which are coupled by the 6 × 6 stiffness matrix *Q*. Each dinucleotide has its own intrinsic values and stiffnesses that are fully parametrized in the literature [13, 14]. We use here the hybrid parametrization [15] in which intrinsic deformations are derived from protein-DNA crystals and the stiffnesses from atomistic molecular simulations. We note that the results are rather robust with respect to the specific choice of parametrization: the main conclusions of this paper also hold for the Olson-parametrization [13] (data not shown).

The DNA is forced into a superhelix by constraining the positions and orientations of 28 middle-frames of consecutive bp that correspond to DNA phosphates bound to the histone octamer. We identified these 28 strongly bound phosphates from local minima in the crystallographic B-factor in the NCP147 structure [16]. They give rise to 14 distinct nucleosome binding sites, each containing two bound phosphates. We studied several nucleosome crystal structures and found that each pair of DNA phosphates connecting two successive base-pairs, whether bound to the octamer or not, is always fixed with respect to the middle-frame, the coordinate system whose position and orientation is exactly in between those of the two base-pairs. This allows us to implicitly take bound phosphates into account, even though our model consists only of rigid base-pairs. See S1 Text, S1 and S2 Figs for more details on the construction of the constraints. Compared to other similar models in the literature [17–21], the benefit of our model is that it does not contain free parameters and allows for an efficient Monte Carlo sampling.

It was found in a recent computational study [22] that two main features determine the sequence affinity to nucleosomes: intrinsic curvature and minor groove width. Both features are manifestations of the equilibrium shape of the unbound DNA which is accounted for in the rigid base-pair model by the intrinsic values of the bp steps, the *q*^0^’s in Eq 1. The role of intrinsic curvature to alleviate the cost of bending DNA into nucleosomes is obvious and is accounted for also by other studies like Refs. [18, 20]. The role of the minor groove width is more subtle and is neglected in those studies. Our model, however, automatically takes the minor groove width into account. Each binding site consists of two phosphates where the DNA is fixed to the histone core, one on either side of the minor groove. A mismatch in minor groove width (as prescribed by the equilibrium shape of the DNA) thus automatically leads to a frustrated molecule, increasing the energetic cost.

There are a great many models in the literature that attempt to predict the nucleosome affinity of sequences. These models can be divided into two main classes: bioinformatics models trained on experimental nucleosome maps (see e.g. [1]), and physical models that account for intrinsic DNA elasticity (see e.g. [18]). A systematic comparison of the performance of eight different models is provided in Ref. [23] and an overview over more than 30 different kind of models is listed in Ref. [24]. Due to the detailed nature of our model, it is unfortunately not computationally feasible to perform the whole-genome analyses that are usually performed to test the predictiveness of models. Instead we apply a test similar to the one found in Ref. [22]. We compare the predicted differences in nucleosome formation energy, ΔΔ*G*_model_, between 22 pairs of DNA sequences to the corresponding experimental values, ΔΔ*G*_exp_ (see S3 Fig and S1 Text for details). The root-mean-square deviation between our model prediction and the experimental data is 1.2*k*_*B*_*T*.

### Mutation Monte Carlo method (MMC)

MMC is based on the standard Metropolis algorithm with two types of Monte Carlo moves: spatial moves and mutation moves. The spatial moves change the DNA conformation and are designed such that the constraints on the middle-frames are not violated. The mutation moves change the bp sequence but keep the DNA configuration fixed. Both moves change the energy, Eq (1), of the two bp steps involved. By applying the mutation and configurational moves together, it is possible to move in sequence space as well as in configuration space and to perform an optimization in both spaces simultaneously. A variant of this method, synonymous Mutation Monte Carlo (sMMC), is used on top of genes. In sMMC our mutation step consists of randomly picking a codon and replacing it by a synonymous one. Note that we treat all degrees of freedom (conformation and sequence) on an equal footing, especially that we use one and the same temperature for both conformational and mutation moves. This way we ensure proper sampling at thermodynamic equilibrium. The role of temperature is to create a balance between the binding affinity and the diversity of the sequences in the ensemble. It is in this purely simulation-technical sense that a temperature is employed here; it should not to be confused with the real temperature inside a live cell.

### Nucleosome-amino acid correlation analysis

To test whether multiplexing occurs in real genomes we introduce the nucleosome-amino acid correlation analysis. For the analysis we used nucleosomes from the redundant maps of *Saccharomyces cerevisiae* [25] and, separately, of *Schizosacharomyces pombe* [26] but only those nucleosomes with NCP score/noise ratio larger than 1.5 (37748 nucleosome in *S. cerevisiae* and 229943 in *S. pombe*). Genes were extracted from the genome.ucsc.edu database (table: sgdGene, output format: GTF). We go through all codons (or, for non-coding parts, trinucleotides) and determine their positions inside nucleosomes, if present, in the redundant maps mentioned above. This produces occurrence probabilities of codons (or trinucleotides) along nucleosomes. All synonymous codons (or corresponding trinucleotides) are then lumped together, resulting in probability distributions of “amino acids” along nucleosomes that reflect their preferred rotational settings. Before analyzing these distributions the central 10 bp were left out to remove possible artifacts from the chemical mapping procedure [25]. In addition, 3 bp were removed from the left DNA terminus, and 4 bp from the right terminus, so that the remaining length, 130 bp, is divisible by 10 bp. Discrete Fourier transformations of the various distributions were performed and the Fourier amplitudes plotted.

## Results

### The nucleosome positioning code is mechanical in nature

As a first step we ask whether the nucleosome positioning rules can be explained on the basis of the sequence dependent mechanical properties of DNA. Undeformed B-DNA is well-described by only two non-vanishing degrees of freedom (a 0.34 nm rise along and a ∼36 degrees twist around the axis perpendicular to the bp plates) leading to a straight, twisted bp stack. To produce a bent molecule like DNA in a nucleosome, other degrees of freedom need to be non-zero, most importantly the roll rotation around the long bp axis. Oscillating the roll values with the DNA helical repeat leads to overall bending. Sequences with high affinity to nucleosomes feature TT/AA/TA dinucleotides at negative roll positions (minor groove facing inwards) and GC dinucleotides at positive roll positions (minor groove facing outwards) [1, 3].

The relations between the experimental nucleosome positioning rules and the model parameters of the rigid base-pair description turn out to be not straightforward. For instance, for high affinity sequences GC steps peak at positive role positions, even though the GC step features the smallest intrinsic roll and the second largest roll stiffness (see S4 Fig). We will see, however, that the rigid base-pair model is capable of predicting the nucleosome positioning rules, despite this apparent paradox.

To learn which sequence motifs have small energy costs for wrapping into a nucleosome, we let our bent model DNA freely explore sequence space. This is achieved by performing MMC on our nucleosome model. Fig 1B shows the result of our MMC simulation, which was performed at 100 K. Plotted are the dinucleotide occurrence probababilities (or frequencies) for GC and separately for TT/AA/TA along the nucleosome, derived from an ensemble of 10^7^ sequences.

The size of that subset and the affinity of the sequences can be controlled by the effective temperature of the MMC simulation. This in turn is reflected in the amplitude and sharpness of various dinucleotide distributions. S5 Fig shows as an example the AA distribution produced by MMC for three different temperatures. An overview of the distributions for all dinucleotide steps is provided in S6 Fig. As can be seen, there are several steps that peak around positive roll positions, namely CC, CG, GC and GG, and around negative roll position, namely AA, AT, TA and TT. These are combined in the probability distributions depicted in S7 Fig which provide slightly larger amplitudes than the one including fewer dinucleotide steps in Fig 1B. The same combinations of dinucleotide steps were also presented in experimental studies, see e.g. the work of Kaplan et al. [27].

By adjusting the effective temperature in our simulation we could in principle create via MMC distributions with the same amplitudes as in the experiments but it is important to note that the experimental distributions are biased by the methods that were used to create them. For instance, *in vivo* distributions created by micrococcal nuclease digestion are biased to feature strong signals at the outer peaks reflecting nucleosomes that are protected against partial digestion due to nucleosome breathing, whereas *in vitro* distributions show strong signals for the inner peaks reflecting the salt dialysis reconstitution protocol where the tetramer binds first (see Fig 1b in Ref. [1]). Dinucleotide distributions created from chemically mapped nucleosomes show much sharper signals but might suffer from some biases, especially favoring nucleosomes with an A at position -3 and a T at position +3 with respect to the dyad [25].

Note that we find from our model that high affinity sequences show peaks of GC at positive roll positions, Fig 1B, even though these are energetically the least preferred positions, as mentioned above. The reason that GC occurrences are biased against GC’s own intrinsic preferences is that GC brings in good neighbours. For instance, the tetranucleotide AGCT is energetically favorable because of the AG and CT steps. So even though the mechanical energies in our DNA model are local, see Eq (1), the trivial fact that dinucleotide steps need to be compatible with each other leads to a strong correlation along the sequence that is sufficient to explain the surprising positions of the GC-peaks in Fig 1B. For a detailed discussion of the positioning rules we refer the interested reader to S2 Text, S8 and S9 Figs. Altogether our findings show that a model based on DNA mechanics alone is capable of predicting the basic nucleosome positioning rules.

### Genetic and mechanical information can be multiplexed

We next ask ourselves whether nucleosomes can be also positioned on top of genes. As a first test we redo the simulations from the previous section but this time on top of coding sequences. Will we recover the same positioning rules in this case? We introduce a variant of the MMC method, the synonymous Mutation Monte Carlo method (sMMC). As a nucleosome sits on a gene at a specific position, its wrapping sequence can be considered as a sequence of 49 codons. A mutation move consists now of picking a random codon, replacing it by a synonymous codon (possible for 18 of the 20 amino acids) and then accepting or rejecting the mutation according to the energy change.

When performing sMMC on a given coding sequence, the dinucleotide occurrence probabilities show extremely sharp peaks at various positions (result not shown), reflecting fixed dinucleotide steps. However, averaging this procedure over 1200 random codon sequences, we recover the positioning rules, albeit with a smaller amplitude (Fig 1C). The degeneracy of the genetic code thus allows to put mechanical signals on top of genes, but to what extent?

**Fig 2:**
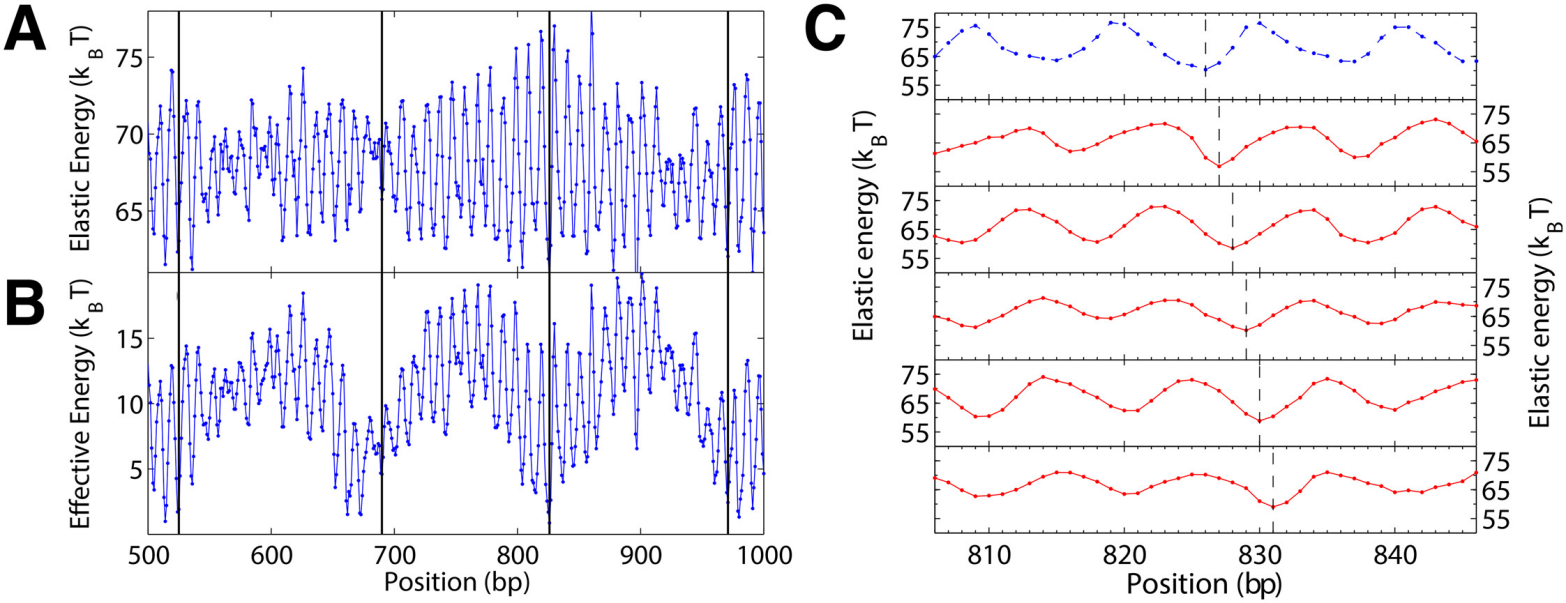
Mechanical energy landscape along a 500 bp stretch of the YAL002W gene of *S. cerevisiae*. (A) Elastic energy of the nucleosome model as a function of position obtained from a Monte Carlo simulation at 50 K. (B) Effective energy including excluded volume between nucleosomes. In both, (A) and (B), the vertical lines indicate experimentally determined nucleosome positions from the unique nucleosome map [25]. (C) The top graph shows a fraction of the original landscape from (A), the five landscapes below are produced via sMMC with the nucleosome positioned at the corresponding dashed vertical line. The minima can be shifted freely on top of genes, proving that multiplexing is possible.

To answer this question we pick a random gene, YAL002W of *S. cerevisiae*. Fig 2A shows the energy landscape of a 500 bp long stretch along that gene (see S10 Fig for the whole gene). For each nucleosome position we measure the energy by performing a Monte Carlo simulation at low temperature. The ∼10 bp periodic undulations in the resulting landscape reflect a preferred local bending direction of the involved DNA stretch. The vertical lines in the plot indicate *in vivo* positions of nucleosomes that have been mapped with bp resolution [25]. Positions of local minima are typically very close to mapped nucleosomes, indicating that the rotational positioning of nucleosomes *in vivo* is mainly determined by the DNA molecule itself (see also S10, S11 Figs and S1 Text). The prediction of the translational positioning improves when we account for excluded volume between nucleosomes (see Fig 2B, S10 Fig and S1 Text).

We can now define what we mean by multiplexing classical genetic and mechanical information: the ability to shift the local minima at will without changing the encoded protein sequence. To test whether this is possible we pick the deep minimum at position 826 with respect to the beginning of the gene which coincides with a unique nucleosome in the experimental map (Fig 2C top). We then attempt to create a minimum at a new location by putting the nucleosome at the desired position (indicated by dashed lines in Fig 2C) and performing sMMC. We find that we can create a local minimum anywhere without altering the gene, even at a previously highly unfavourable position, demonstrating the possibility of multiplexing of genetic and mechanical information. Therefore, our findings suggest that nucleosomes can be positioned anywhere on top of genes.

### Three mechanisms underlie multiplexing

The late Jonathan Widom asked in one of his last talks [28] how classical and mechanical information can be multiplexed and claimed that there are “three non-exclusive answers” to that question. We will cite each point verbatim and test it with our model.

**Fig 3:**
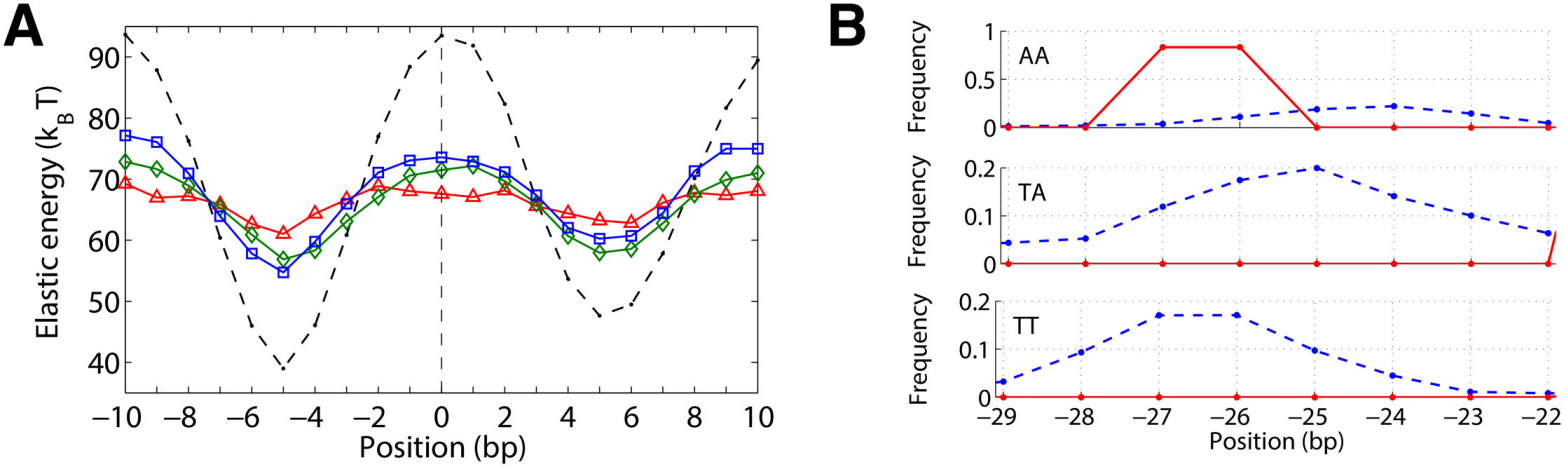
Mechanisms underlying multiplexing. (A) Energy landscape (black dashed curve) of a sequence with highly optimized nucleosome affinity at −5 bp, produced by MMC at very low temperature (15 K). The colored curves are landscapes for three synonymous mutants (three different codon frames) that are optimized via sMMC for high affinity at position 0. The maximum cannot be turned into a minimum in this case, signaling that multiplexing would not be possible on genomes if they were selected for highest nucleosome affinity. (B) Distribution of AA, TT and TA dinucleotides around minor groove bending site −25 bp for the shifted nucleosome from Fig 2C bottom. Dashed blue curves: natural preferences (attained through MMC), red curves: distribution obtained from sMMC on that particular stretch of the YAL002W gene; both simulations are performed at 100 K. Though not optimal, sMMC brings in AA at a position close to its preferred position (amplitude almost 1) indicating the plasticity of the mechanical code.

The first reason is that no region of a genome “is selected for highest possible nucleosome affinity. (…) So there is a tremendous sequence degeneracy, because the goal seems not to be highest possible affinity.” To illuminate this idea we create a sequence with very high affinity, by performing MMC and decreasing the temperature to very low values. The resulting sequence is then extended by forming a tandem repeat and the nucleosome is moved along it. As can be seen in Fig 3A, the elastic energy landscape (dashed black curve) shows very large undulations, much larger than the ones observed for the YAL002W gene, Fig 2A. The deep minimum at position −5 bp represents the generated high-affinity sequence. We position our nucleosome at the maximum of the energy landscape, 5 bp to the right, at 0 bp. We assume now that the wrapped sequence is a coding sequence and that the direction of transcription is from the left to right. This leaves three possible codon frames. For each frame we attempted to create a new minimum at this position by performing sMMC at low temperatures. The resulting energy landscapes (three colored curves in Fig 3A) show a reduction in amplitude but not a shift in the positions of the maximum and minimum. In short, if sequences were selected for highest possible nucleosome affinity, multiplexing would not work.

Secondly, “protein coding sequences are highly degenerate.” This degeneracy underlies our sMMC scheme; in fact it has been argued that the actual genetic code is better than most other possible genetic codes to support multiplexing [29].

Finally, there is “a beautiful feature of DNA mechanics that allows (…) a good motif like a TA step (…) to be used at a suboptimal location, for example a base-pair to the left or right, and being nearly as good as it were in the optimal location.” This feature plays also a role in our simulations. Take for example our exercise from the previous section, where we displaced a well-positioned nucleosome on the YAL002W gene to a new position 5 bp to the right (Fig 2C). At position −25 from the dyad, we have the minor groove facing inwards, so the nucleosome would like to have an AA/TT/TA step here. However, this dinucleotide is AC, the first of a codon that encodes for threonine. All codons that encode for threonine start with AC, so this dinucleotide is locked into this suboptimal configuration. At position −26, however, there is some freedom: this is the step connecting the last nucleotide of a codon that must encode for glutamic acid (a G or an A), and the first nucleotide of the threonine codon (an A). This allows for two possible steps, either AA or GA. We find that sMMC brings in AA most of the time, even though −26 is normally a less strongly preferred position for this dinucleotide, see Fig 3B.

To summarize, our model corroborates Widom’s claims: multiplexing works because of sequence properties of genomes, the degeneracy of the genetic code and the plasticity of the mechanical code.

### Real genomes multiplex genetic and mechanical information

The analysis of the previous three sections shows that it is in principle possible that DNA molecules carry purely mechanical information along their sequences, and that these signals can be multiplexed with genetic information. These two types of information would be the result of parallel and independent evolutions. However, it could very well be that there is not enough selective pressure for a mechanical evolution to take place on large length scales and that it only affects localized stretches like transcription start and translation end sites [27]. That the experimentally mapped nucleosomes on top of genes show strong signals in the dinucleotide occurrence probabilities [1, 3, 25] does not prove by itself the presence of mechanical signals since similar distribution can also be produced by systematically shifting nucleosomes on random sequences [30]. In that recent publication the fraction of nucleosome sequences containing statistically significant 10.5 bp periodic signals in dinucleotides was estimated to be only about 3%. Given the smallness of such signals, it is to be expected that it is hard to unequivocally isolate signals for mechanical cues on top of genes. This problem is compunded by the presence of various perturbing factors. To give a few examples: There is a relation between codon usage and co-translational folding via the translation elongation rate [31] that might be of importance. Codon usage biases have been shown to be similar for genes that are typically close to each other in 3D space [32]. Mutation rates on DNA stretches wrapped in nucleosomes can be higher [33] or lower [34] than for linker DNA.

It is thus interesting to approach the problem whether genome sequences have evolved to position nucleosomes from a different angle. We try to minimize the influence of perturbing effects like the ones mentioned above by asking: Is there any relation between the bp sequences of genomes and the positions of mapped nucleosomes that could hint at multiplexing? Consider first a scenario without multiplexing. A nucleosome on top of a gene would sit typically in a local energy minimum, a stretch of gene that accidentally conforms with the dinucleotide positioning rules. We can also look at the corresponding rules of trinucleotides [3] or—as we are on top of a gene—we can lump all synonymous codons together leading to mechanical rules for amino acids. A given amino acid (i.e. its set of synonymous codons) typically shows preferences for either positive or negative roll positions. Now assume that there is an evolutionary advantage that a substantial fraction of those nucleosomes is shifted to new positions (like we shifted a nucleosome on top of the YAL002W gene in Fig 2C). This can be achieved by subtle position dependent biases in the synonymous codon usage. As nucleosomes are shifted, their correlations with amino acid positions are lost, lowering the amplitudes of the periodic signals of amino acids.

It is not possible to test this idea directly but we can compare the statistical features between coding and non-coding sequences. To do so we look at the distribution of trinucleotides along nucleosomes in non-coding regions and lump them together to virtual “amino acids.” We expect that there is less information and multiplexing present in non-coding regions and that the resulting distributions reflect closer the mechanical preferences of the “amino acids” than coding regions do.

**Fig 4:**
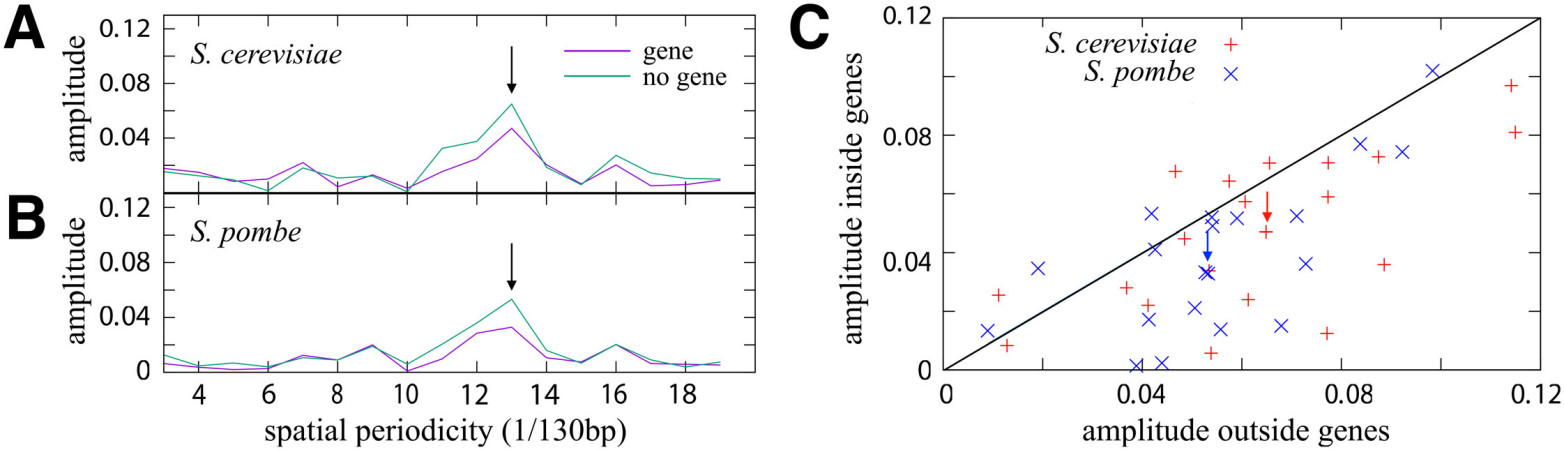
Multiplexing in two eukaryotic genomes. (A) Normalized Fourier amplitudes for the distribution of the synonymous codons for threonine along nucleosomes on top of genes (purple curve) and for the distribution of the corresponding trinucleotides along nucleosomes outside genes (green curve) [25]. The peaks at 10 bp (indicated by an arrow) are due to nucleosome positioning that appears weaker on top of genes but might signal multiplexing instead. (B) Same as (A), but for *S. pombe* [26]. (C) The normalized 10 bp amplitude inside vs. outside genes of all 20 amino acids for the two yeast species. The arrows indicate threonine. All points below the line have smaller amplitudes inside genes, a hallmark of multiplexing.

Fig 4A shows the normalized Fourier amplitudes of the probability distributions for the amino acid threonine along nucleosomes on top of coding and non-coding regions of the *S. cerevisiae* genome [25] (see Methods and Models for details; S12 Fig displays the Fourier amplitudes of all amino acids). Both spectra show a peak at 10 bp, indicating that threonine codons have an overall rotational preference with respect to the DNA bending inside nucleosomes. Most importantly, the amplitude for the non-coding peak is significantly higher than the peak for coding sequences. The same is true for *S. pombe*, Fig 4B, for which a high resolution nucleosome map also exists [26]. In Fig 4C we plot the amplitude of the 10 bp periodicity for coding versus the corresponding non-coding values for all 20 amino acids in the two organisms. The majority of the points is found in the lower right triangle suggesting that multiplexing might indeed occur. This is remarkable in view of the fact that in both organisms the large 10 bp amplitudes of the combined dinucleotide steps AA/TT/AT/TA are slightly stronger on top of genes (see e.g. Fig 2A in Ref. [26] for a comparison between exons and introns in *S. pombe*). The strength of this effect (e.g. the percentage of nucleosomes shifted on top of genes) is, however, hard to gauge since systematic differences between coding and non-coding regions might exaggerate or weaken this effect.

## Discussion

Using a computational nucleosome model we have shown that the major features of the nucleosome positioning rules can be predicted by the sequence dependent DNA geometry and elasticity. We furthermore gave evidence that nucleosomes can be positioned along DNA at arbitrary positions with single bp precision, even on top of genes. Multiplexing of mechanical signals and genetic information is possible due to the degeneracy of the genetic code (and the fact that genomes have not evolved for highest nucleosome affinity). Analysis of two high resolution nucleosome maps revealed strong signals that—even though they do not constitute a definite proof—are at least consistent with such a view. Taken together, all these findings suggest the intriguing possibility that nucleosome positions are the product of a mechanical evolution of DNA molecules.

Here we focused entirely on the nucleosome positioning capability of mechanical information. This is only one aspect of a much wider range of mechanical effects in nucleosomes. The space of ∼10^88^ wrapping sequences hosts a large range of nucleosomes: nucleosomes that adsorb over- or undertwist via small twist defects (similar to the one observed in a crystal structure [4]), nucleosomes that expose their DNA through thermally induced unwrapping, or hide it, or nucleosomes that unwrap under force along a prescribed path, possibly in a highly asymmetric fashion (recently observed for the 601 sequence [35]). The latter might be important for the interaction with an elongating RNA polymerase [36]. There might be also nucleosomal sequences that are highly sensitive to CpG methylations [37], forming hotspots for a mechanical epigenetics.

Nucleosomes can thus be considered as a highly diverse class of DNA-protein complexes with a near continuous range of physical properties. Special nucleosomes could be designed *in silico* with our MMC method and their properties probed via *in vitro* experiments. It will be interesting to study whether well-positioned nucleosomes with special properties reflecting their genomic context have also emerged through a mechanical evolution.

## Supporting Information

S1 Text Model and methods. (PDF)

S2 Text Interpretation of the positioning rules for the model nucleosome. (PDF)

S1 Fig Base-pair step together with its corresponding midframe. The red spheres represent the phosphates whose positions with respect to the middle frame are given by Eqs (2) and (3) in S1 Text.
(PDF)

S2 Fig DNA phosphate positions inside nucleosome crystal structures. The distribution functions of *a*, *b* and *c* as defined in Eq (2) of S1 Text, for all the phosphates in the NCP147 [16] (red) and NCP601L [38] (green) crystal structures.
(PDF)

S3 Fig Predicted and experimental binding free energy. Each point corresponds to a pair of DNA molecules, 22 pairs in total: c1/c2, c1/c3, d1/d2, d1/d3, d1/d4, d1/d5, e1/e2, e1/e3 [1], TG/TG-T, TG/TR-5, TG/TRGC [42], TG/ANISO, TG/TTT, TG/NOTA, TG/EXAT, TG/EXGC, TG/IAT, TG/IGC, TG/END, TG/ANNA, TG/34 and TG/20 [43]. The dashed line corresponds to perfect agreement. The root-mean-square deviation between our model prediction (the tetramer free energy; see S1 Text for detail) and the experimental data is 1.2*k*_*B*_*T*.
(PDF)

S4 Fig (A) The tilt stiffness versus the intrinsic tilt and (B) the roll stiffness versus the intrinsic roll for the ten distinct dinucleotide steps in our model. For the remaining six steps, the bending parameters are simply obtained by the inversion transformation, which changes the sign of the intrinsic tilt and keeps other parameters unchanged.
(PDF)

S5 Fig Dependence of dinucleotide distributions on effective temperature. Probability distribution of the AA step, obtained by the MMC for three different temperatures: *T* = 600 K (blue), *T* = 100 K (green) and *T* = 21 K (red).
(PDF)

S6 Fig A color-map of the frequencies for all 16 dinucleotide steps as a function of the position. The distributions are obtained in a Mutation Monte Carlo simulation at temperature 100 K.
(TIFF)

S7 Fig Nucleosome positioning rules. (A) Fraction of dinucleotides AA/AT/TA/TT and separately CC/CG/GC/GG at each position along the nucleosome model found in 10 million high affinity sequences produced by MMC at 100 K. The model recovers the basic nucleosome positioning code. (B) Same as (A) but on top of 1200 randomly generated coding sequences (produced by sMMC). The same periodic signals are found albeit with a smaller amplitude.
(PDF)

S8 Fig Comparison between model and crystal structure. The averaged degrees of freedom for NCP147 DNA sequence as obtained in the model (solid curves, blue), in comparison with the crystal structure (dashed curves, red) [16].
(PDF)

S9 Fig The occurrence frequencies of TTAA (triangles, red) and AGCT (dots, green) as obtained in an unconstrained Mutation Monte Carlo simulation at 100 K. The solid and dashed vertical lines indicate minor and major groove bending sites respectively.
(PDF)

S10 Fig Translational positioning of nucleosomes. The effective energy landscape with *μ* = 80*kT* (red curves), the elastic energy landscape (blue curves) and the experimentally mapped nucleosomes [25] (vertical black lines) along the YAL002W yeast gene. The elastic energy is shifted down by 30*kT* for clarity. The top panel shows the landscapes over the entire gene. Each of the remaining panels zooms into a 765 bp long portion of the gene. All of the experimentally mapped nucleosome positions fall into local minima. In addition, the corresponding minima are quite deep in the central region of the gene.
(PDF)

S11 Fig Rotational positioning of nucleosomes. (A) The histogram of the distances between 1293 experimentally mapped nucleosomes [25] on yeast chromosome I and the nearest local minima in the theoretical energy landscape (red rectangles). As a comparison we show also the prediction from a probabilistic model trained on *in vitro* data (blue rectangles) [27]. (B) The distance histogram as defined in (A) for 769 nucleosomes on yeast chromosome I which are located on the genes. The two histograms are quite similar. In both cases, 60 percent of the experimental nucleosome positions lie within the range of one bp around a local minimum in the theoretical energy landscape.
(PDF)

S12 Fig Evidence for multiplexing in two eukaryotic genomes. Normalized Fourier amplitudes of the distribution of the synonymous codons for all 20 amino acids along nucleosomes on top of genes (purple curve) and of the distribution of the corresponding trinucleotides along nucleosomes outside genes (blue curve) for *S. cerevisiae* (left) and *S. pombe* (right). The peaks at spatial periodicity 13 corresponds to a 10 bp periodic signal. In most cases the height of this peak is larger for the non-coding case, evidence for multiplexing of genetic and mechanical information.
(PDF)
