# Supplementary material for: Multiplexing Genetic and Nucleosome Positioning Codes: A Computational Approach
Source: PLoS One. 2016 Jun 7;11(6):e0156905. doi: 10.1371/journal.pone.0156905 (PMC4896621; doi:10.1371/journal.pone.0156905)
Supplement: S1 Text — (PDF) [file pone.0156905.s001.pdf]

S1 Model and methods

Constructing the constraints: The main DNA-histone interactions are hydrogen bonds that involve 28 distinct DNA phosphates [4]. However, the rigid base-pair model does not contain the phosphates explicitly. Here we determine their positions with respect to the middle-frame of the corresponding basepair (bp) steps. The position vector of each phosphate along the DNA with respect to the corresponding middle-frame (see S1 Fig) can be written as

$$\mathbf{r}_{\mathrm{P}} = a_i \hat{\mathbf{d}}_1^m + b_i \hat{\mathbf{d}}_2^m + c_i \hat{\mathbf{d}}_3^m \qquad i = 3,5. \tag{2}$$

In this equation $\hat{\mathbf{d}}_1^m$, $\hat{\mathbf{d}}_2^m$ and $\hat{\mathbf{d}}_3^m$ are the basis vectors of the middle-frame, and a, b and c are the components of the relative position vector along these basis vectors, with the subscripts 3 and 5 indicating the phosphates on the 3' and 5' strands respectively. S2 Fig shows the distribution functions of a, b and c for all the phosphates in two nucleosome crystal structures: NCP147 [16] and NCP601L [38].

It can be seen that the two crystal structures give rise to similar distributions, and each distribution has a sharp peak. Calculating the average of each distribution for both crystal structures, we obtain

$$\begin{aligned} \langle a_3 \rangle &= \langle a_5 \rangle = -0.30\ \mathrm{nm} \\ \langle b_3 \rangle &= -\langle b_5 \rangle = 0.89\ \mathrm{nm} \\ \langle c_3 \rangle &= -\langle c_5 \rangle = -0.05\ \mathrm{nm}. \end{aligned} \tag{3}$$

The standard deviation of all the distribution functions is about $\sigma \approx 0.05$ nm, comparable to the error in the crystallography experiments [19]. Therefore, within the accuracy of the experimental data, it can be assumed safely that all of the phosphates are firmly attached to the middle-frames and their relative positions are given by the Eqs. (2) and (3).

To identify the strongly bound phosphates in the nucleosomal DNA, we looked at the crystallographic B-factor for all the phosphates in the NCP147 structure [16]. Each local minimum in the B-factor corresponds to a strongly bound phosphate. This gives rise to 14 distinct nucleosome binding sites, each containing two bound phosphates [39]. Starting from the NCP147 crystal structure, we calculated the positions of the bound phosphates from Eqs. (2) and (3) and we assumed that they are all fixed in their places. Since the preferred orientations of the bound middle-frames are in principle affected by the DNA sequence, we do not use the crystal structure to constrain them. Ideally one would derive the elasticities of those constraints from a collection of many nucleosome crystal structures but the currently available data make such an approach unattainable [40]. Instead we allow for a pre-relaxation of the system before fixing the bound middle-frames permanently. The pre-relaxation was performed using the standard Monte Carlo simulation with a homogeneous DNA sequence, to remove any sequence-dependent bias. The configurational Monte Carlo moves during the pre-relaxation course are designed such that they keep the positions of the bound phosphates fixed, but allow for the free rotation of the bound middle frames. After the pre-relaxation we put each of the bound middle-frames in its preferred orientation, and we kept it fixed during all subsequent Monte Carlo simulations.

Nucleosome binding free energies: It is known that nucleosome assembly *in vitro* is initiated by binding the H3-H4 tetramer to the DNA molecule. It is only after the reduction of the salt concentration that the H2A-H2B dimers bind to the tetramer

to form a full nucleosome. Therefore it is expected that the apparent *in vitro* nucleosome free energies are mainly determined by the interaction of the DNA with the histone tetramer [1, 41]. To account for this, we model the tetramer by allowing the DNA to only attach to the ±2.5, ±1.5 and ±0.5 binding sites. S3 Fig shows the binding free energy difference between pairs of DNA molecules (of varying lengths, sometimes even substantially shorter than 147 bp). We calculate the binding free energy accounting for all possible positions of the tetramer on a given DNA molecule. The following 22 pairs were studied: c1/c2, c1/c3, d1/d2, d1/d3, d1/d4, d1/d5, e1/e2, e1/e3 [1], TG/TG-T, TG/TR-5, TG/TRGC [42], TG/ANISO, TG/TTT, TG/NOTA, TG/EXAT, TG/EXGC, TG/IAT, TG/IGC, TG/END, TG/ANNA, TG/34 and TG/20 [43]. The root-mean-square deviation between our model prediction and the data is 1.2 k_BT. This can be compared to the prediction of the computational nucleosome model by de Pablo and coworkers [22] where a subset of our pairs was studied. The root-mean-square deviation of those 9 data points is worse, 2.1 k_BT. This does not necessarily mean that their model is worse, as they considered the free energy of the full nucleosome (at a fixed position). In fact, linear least squares gives for our model $\Delta\Delta G_{\text{model}} = 1.50\,\Delta\Delta G_{\text{exp}} - 0.88\,k_B T$ whereas the full octamer prediction in Ref. [22] shows a much steeper dependence: $\Delta\Delta G_{\text{model}} = 2.20\,\Delta\Delta G_{\text{exp}} - 2.04\,k_B T$.

Nucleosome positioning in vivo: We calculated the energy landscape for a single nucleosome on yeast chromosome I and compared the distances between *in vivo* mapped nucleosomes [25] to local minima in our landscape. A histogram of the distances from the positions of all the 1293 mapped nucleosomes to the nearest local minimum as predicted by our model (more precisely, the smallest energy within a window of 11 bp length centered around each mapped nucleosome) is given in S11 Fig A (red rectangles). About 60 percent of the nucleosomes lie within the range of one bp around a local minimum. As a comparison we show also the prediction from a probabilistic model trained on *in vitro* data (blue rectangles) [27] where about 43 percent of these nucleosomes are within one bp around a local minimum. We found also about 60 percent agreement when restricting this analysis to the 769 mapped nucleosome on top of genes, S11 Fig B. If the nucleosomes were randomly positioned (e.g. if the action of chromatin remodellers would overrule sequence preferences) one would find that only $3 \times 100/11\% \approx 27\%$ of the nucleosomes to be within one bp around a local minimum. As we find a much higher fraction, this demonstrates that – even *in vivo* – a large fraction of nucleosomes is rotationally positioned and that our model is capable of capturing this effect to a substantial extent.

However, a precise comparison with the *in vivo* data not only requires an accurate nucleosome positioning energy landscape, but it also needs to take into account exclusion between nucleosomes that compete for the same DNA substrate. This can be taken care of using statistical physics approaches [44-46], allowing us to calculate the nucleosome density profile along the DNA. Assuming that nucleosomes interact with each other via a hard-core potential, the nucleosome density on a DNA molecule, ρ, as a function of the nucleosome starting point x, satisfies the Percus equation [47]:

$$\mu = E(x) + \ln\rho(x) - \ln\left[1 - \int_x^{x+\sigma} \rho(x')dx'\right] + \int_{x-\sigma}^{x} \frac{\rho(x')}{1 - \int_{x'}^{x'+\sigma} \rho(x'')dx''} dx' \qquad (4)$$

where $\sigma = 147$ bp is the nucleosome footprint, $E(x)$ is the elastic energy landscape and μ is the chemical potential, i.e. the free energy gain by the reservoir when a

nucleosome unbinds from the DNA. Eq. (4) can be solved numerically [48] for the nucleosome density. The effective energy can then be calculated from

$$E_{\text{eff}} = -k_B T \ln \rho . \quad (5)$$

For small chemical potentials, nucleosome positioning is mainly governed by DNA elasticity and thus the elastic and effective energy landscapes are similar. As the chemical potential increases, alternating regions with high and low nucleosome density appear along the DNA molecule. We calculated the effective energy for the YAL002W gene, which resides on chromosome I between positions 143709 and 147533, see Fig 2B and S10 Fig. To diminish end effects, we included the two 1000 bp sequences which flank the gene at its two sides on yeast chromosome I. S10 Fig shows in blue the elastic energy landscape and in red the effective energy landscape for $\mu = 80\, k_B T$ which is about $10\, k_B T$ higher than the average elastic energy of random sequences, a value consistent with the findings in Ref. [46]. 25 distinct high-density regions can be easily identified along the gene, where the effective energy landscape is low, a number equal to the number of experimentally mapped nucleosomes [25], shown in S10 Fig as vertical lines. The experimental nucleosome positions fall typically into the local minima of the effective energy landscape, especially for the nucleosomes at the central part of the gene, where there is an impressive agreement between the model and the experimental data. At the terminal regions, on the other hand, we did not find a strong correlation between the experimental data and the model predictions. This discrepancy could reflect the presence of other DNA binding proteins that may affect nucleosome positioning *in vivo* [46].

Supplemental references

38. Chua EYD, Vasudevan D, Davey GE, Wu B, Davey CA. The mechanics behind DNA sequence-dependent properties of the nucleosome. Nucl Acids Res. 2012;40: 6338-6352.
39. Richmond TJ, Davey CA. The structure of DNA in the nucleosome core. Nature. 2003;423: 145-150.
40. Meyer S, Everaers R. Inferring coarse-grain histone-DNA interaction potentials from high-resolution structures of the nucleosome. J. Phys.: Condens. Matter. 2015; 27: 064101.
41. Dong F, van Holde KE. Nucleosome positioning is determined by the $(H3\text{-}H4)_2$ tetramer. Proc Natl Acad Sci USA. 1991;88: 10596-10600.
42. Shrader TE, Crothers DM. Artificial nucleosome positioning sequences. Proc Natl Acad Sci. USA. 1989;86: 7418-7422.
43. Shrader TE, Crothers DM. Effect of DNA sequence and histone-histone interactions on nucleosome placement. J Mol Biol. 1990;216: 69-84.
44. Schwab DJ, Bruinsma RF, Rudnick J, Widom J. Nucleosome switches. Phys Rev Lett. 2008;100: 228105.
45. Chevereau G, Palmeira L, Thermes C, Arneodo A, Vaillant C. Thermodynamics of intragenic nucleosome ordering. Phys Rev Lett. 2009;103: 188103.
46. van der Heijden T, van Vugt JJFA, Logie C, van Noort J. Sequence-based prediction of single nucleosome positioning and genome-wide nucleosome occupancy. Proc Natl Acad Sci USA. 2012;109: E2514- E2522.
47. Percus JK. Equilibrium state of a classical fluid of hard rods in an external field. J Stat Phys. 1976;15: 505- 511.
48. Vanderlick TK, Scriven LE, Davis HT, Solution of Percus's equation for the density of hard rods in an external field. Phys Rev A. 1986;34: 5130-5131.
