# Supplementary material for: Multiplexing Genetic and Nucleosome Positioning Codes: A Computational Approach
Source: PLoS One. 2016 Jun 7;11(6):e0156905. doi: 10.1371/journal.pone.0156905 (PMC4896621; doi:10.1371/journal.pone.0156905)
Supplement: S2 Text — (PDF) [file pone.0156905.s002.pdf]

S3 Interpretation of the positioning rules for the model nucleosome

We used the Monte Carlo simulation in the configuration space to find the averaged DNA structure in our model nucleosome. S8 Fig shows the averaged degrees of freedom for the NCP147 DNA sequence as obtained in the model (solid curves, blue), in comparison with the crystal structure [16] (dashed curves, red). As can be seen, the rotational degrees of freedom are clearly correlated with the crystal structure. In particular, the model captures the periodic variations in roll. However, the large variations in shift and rise are not reproduced by the model, and the peaks in the slide are underestimated. Despite these shortcoming our model is successful in predicting the positioning rules that are discussed here in more detail.

The emergence of the nucleosome positioning rules in our model is connected with DNA bending. In the rigid base-pair model the DNA bending is expressed in terms of two perpendicular bending modes known as tilt and roll. Tilt corresponds to the bending of a dinucleotide step over its backbone, while positive and negative rolls are defined as bending toward the major and minor grooves respectively. As mentioned above, bending the DNA into a super-helical configuration results in periodic oscillations of roll and tilt in our model nucleosome, see S8 Fig. These oscillations have a period of 10 bp and a phase difference of 2.5 bp approximately. Roll has a minimum at the minor groove bending sites, while tilt changes sign from positive to negative. The opposite occurs at the major groove bending sites, where roll reaches its maximum value and tilt goes from negative values to positive values.

We found that the nucleosome positioning rules in our model either make DNA locally softer with respect to bending, or help the DNA to intrinsically bend into the correct ”direction” at the minor groove and major groove bending sites. As a typical example, S9 Fig shows the occurrence frequencies of two tetranucleotides along the nucleosomal DNA, namely TTAA and AGCT.

As can be seen, TTAA prefers the minor groove bending sites while AGCT prefers the major groove bending sites. This can be understood by looking at the tilt and roll elastic parameters in the model (S4 Fig). Although the TA step has a relatively large positive intrinsic roll, it is the softest step with respect to bending. On the other hand AA and TT steps are rather rigid, but they have the lowest intrinsic roll after GC, and also have significantly high negative and positive intrinsic tilts respectively. Therefore the motif TTAA is suitable for the minor groove bending sites. Considering the motif AGCT, one can see in S4 Fig that the GC step is one of the most resistant steps towards bending. In addition AG/CT steps are rather stiff. Nevertheless AGCT occurs at the major groove binding sites because AG/CT steps have large positive intrinsic roll and the highest negative and positive intrinsic tilts respectively, so the intrinsic bending of AGCT is compatible with the DNA bending at these locations.

Similar situations occur for CG, CC, and GG steps, as these steps can come together as neighbours of the GC step, and they all provide a favorable intrinsic bending for DNA at the major groove bending sites (S4 Fig). Furthermore, CG is the softest step with respect to bending after TA. As another minor groove example, the AT step has low intrinsic roll and zero intrinsic tilt, and is much stiffer compared to TA. At high enough temperatures, it appears in the Mutation Monte Carlo simulation because it can act as a bridge between an AA or a TA step at the left, and a TT or a TA step at the right.

The above examples illustrate how the nucleosome positioning rules can be understood.
