# Supplementary figures and images for: Multiplexing Genetic and Nucleosome Positioning Codes: A Computational Approach
Source: PLoS One. 2016 Jun 7;11(6):e0156905. doi: 10.1371/journal.pone.0156905 (PMC4896621; doi:10.1371/journal.pone.0156905)

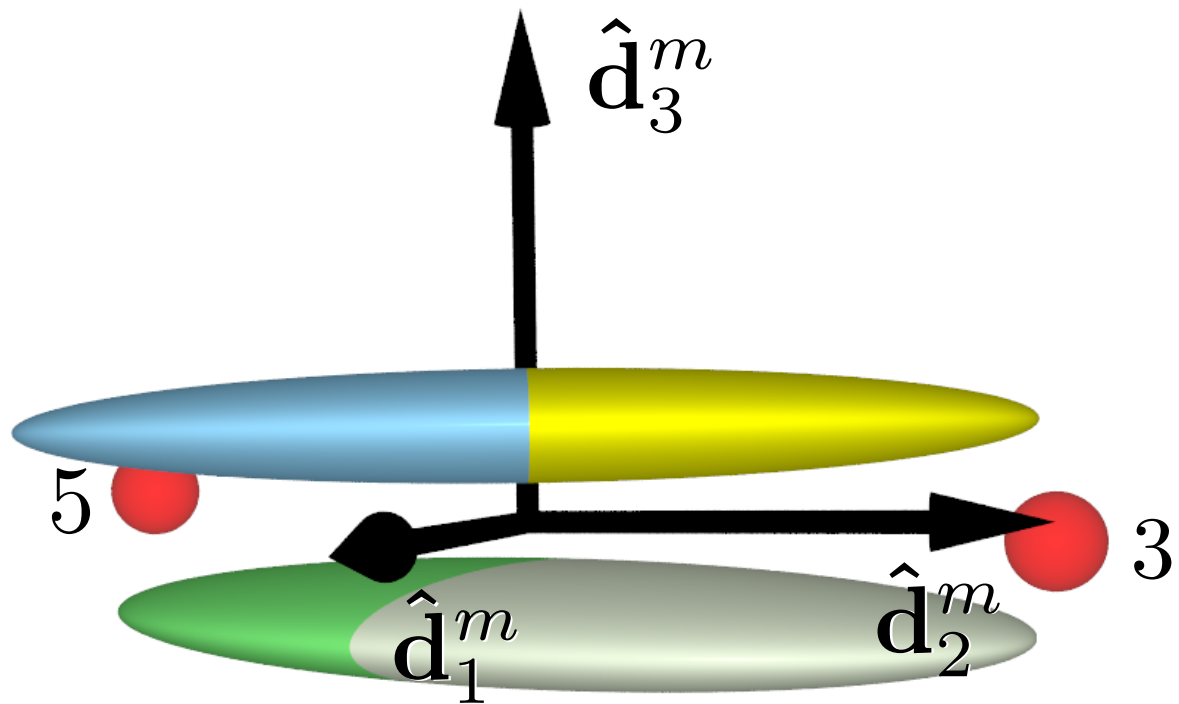

Supplement: S1 Fig — The red spheres represent the phosphates whose positions with respect to the middle frame are given by Eqs (2) and (3) in S1 Text. (PDF) [file pone.0156905.s003.pdf]

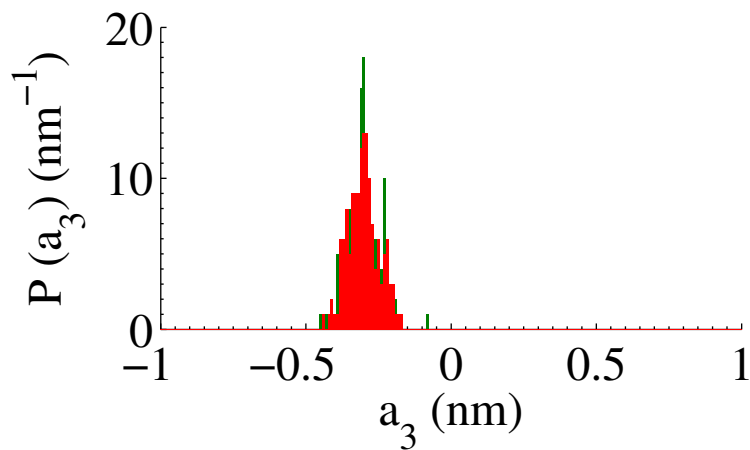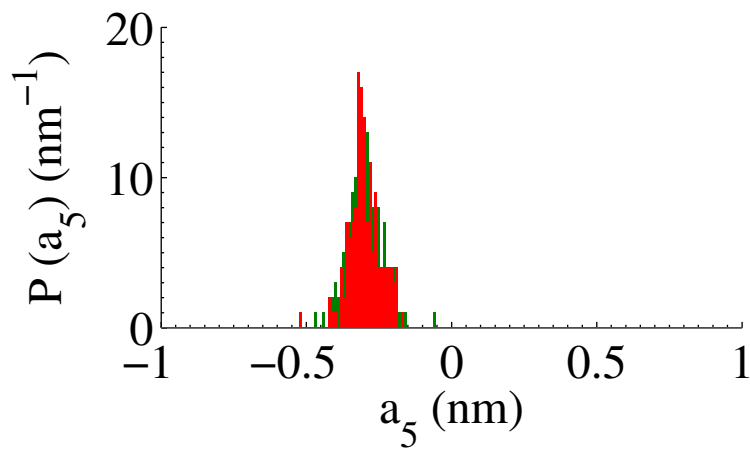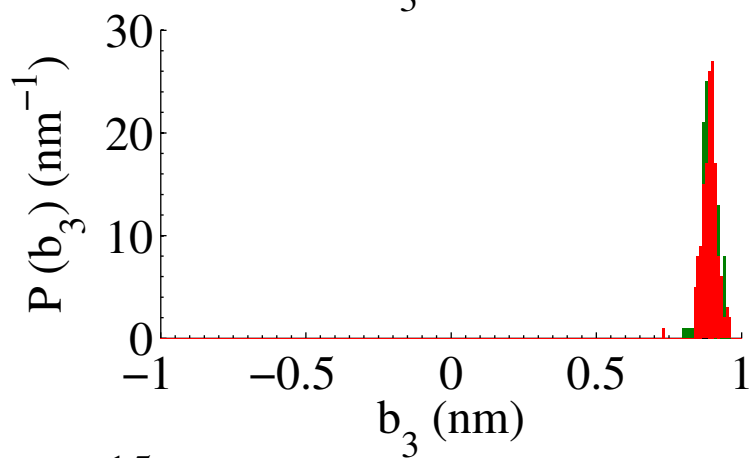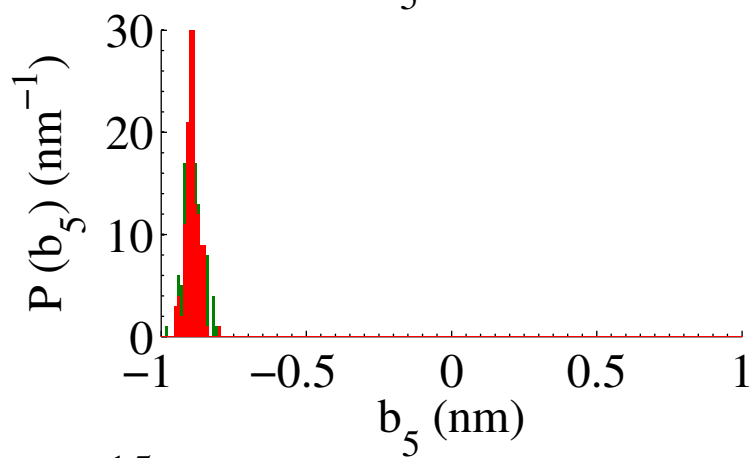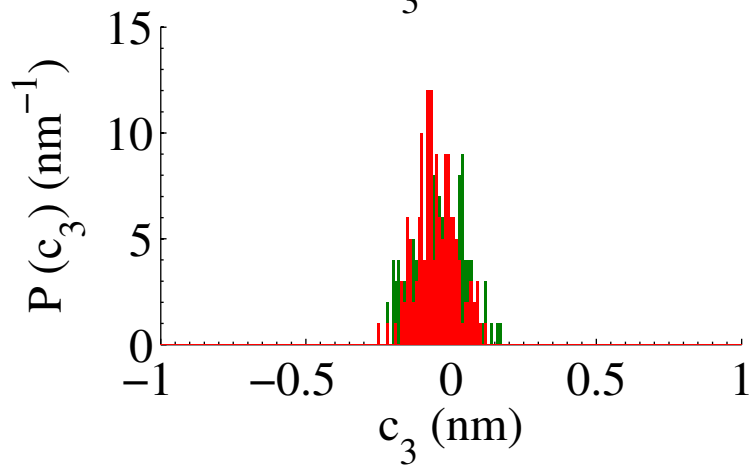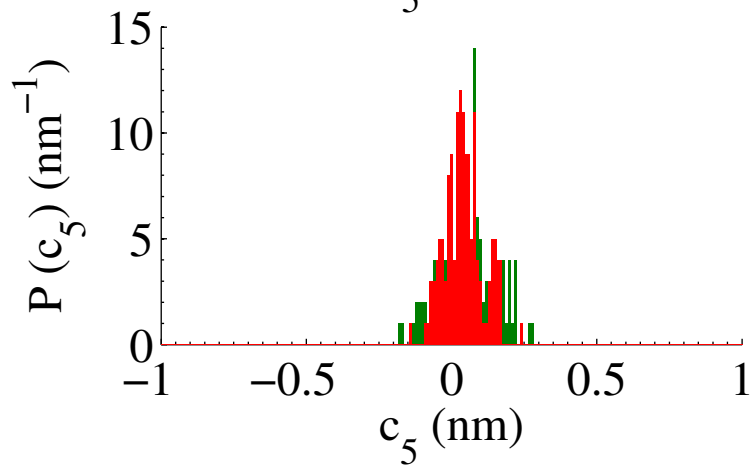

Supplement: S2 Fig — The distribution functions of a, b and c as defined in Eq (2) of S1 Text, for all the phosphates in the NCP147 [16] (red) and NCP601L [38] (green) crystal structures. (PDF) [file pone.0156905.s004.pdf]

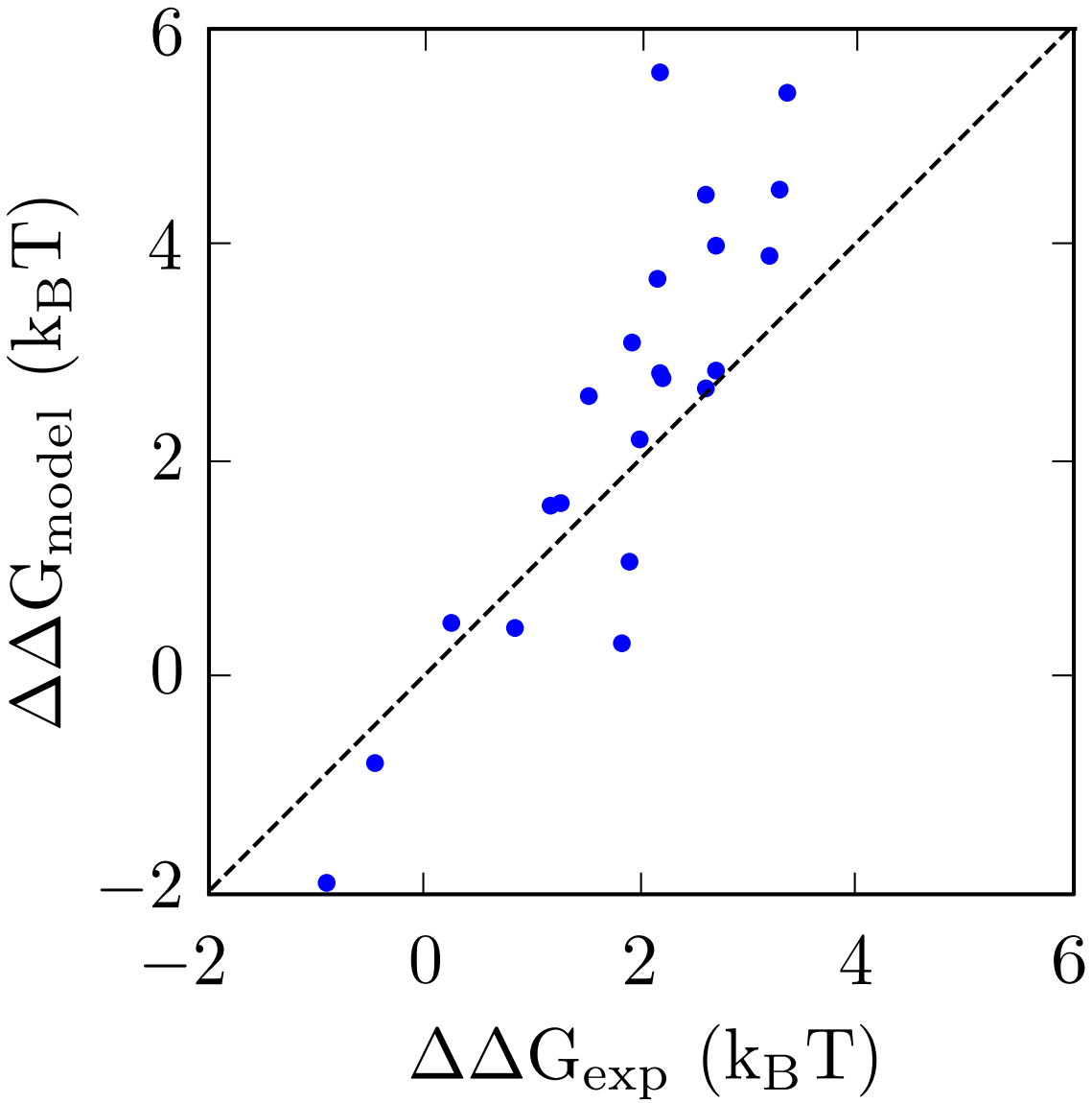

Supplement: S3 Fig — Each point corresponds to a pair of DNA molecules, 22 pairs in total: c1/c2, c1/c3, d1/d2, d1/d3, d1/d4, d1/d5, e1/e2, e1/e3 [1], TG/TG-T, TG/TR-5, TG/TRGC [42], TG/ANISO, TG/TTT, TG/NOTA, TG/EXAT, TG/EXGC, TG/IAT, TG/IGC, TG/END, TG/ANNA, TG/34 and TG/20 [43]. The dashed line corresponds to perfect agreement. The root-mean-square deviation between our model prediction (the tetramer free energy; see S1 Text for detail) and the experimental data is 1.2kBT. (PDF) [file pone.0156905.s005.pdf]

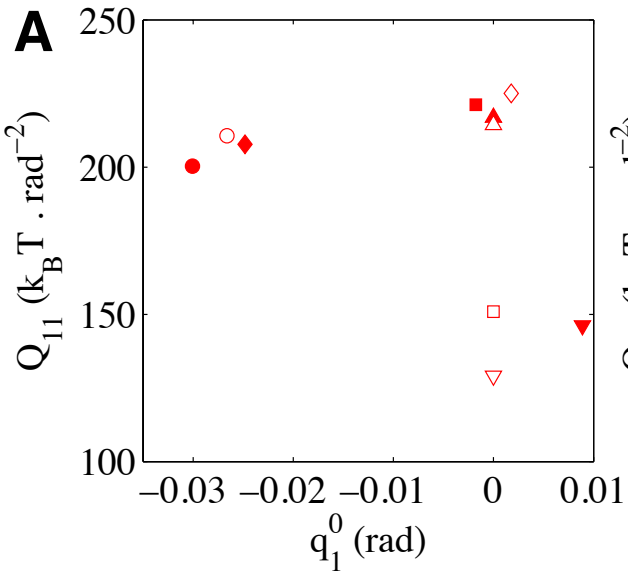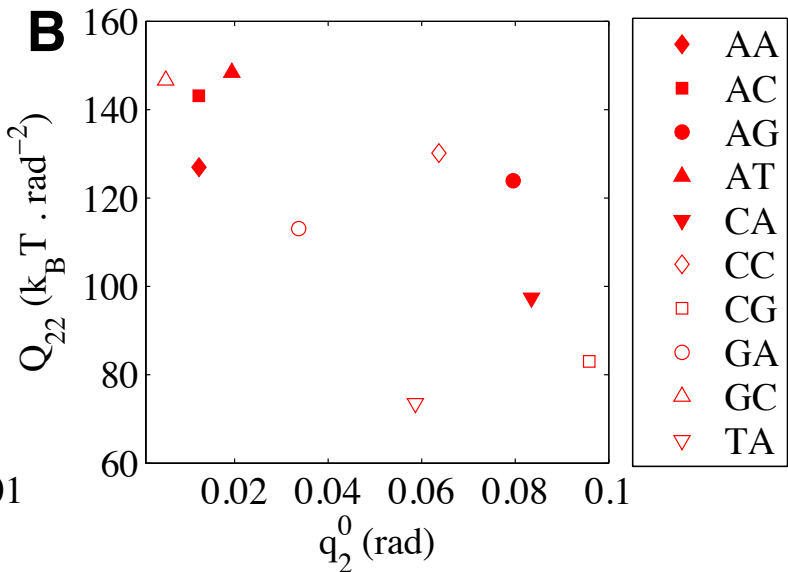

Supplement: S4 Fig — For the remaining six steps, the bending parameters are simply obtained by the inversion transformation, which changes the sign of the intrinsic tilt and keeps other parameters unchanged. (PDF) [file pone.0156905.s006.pdf]

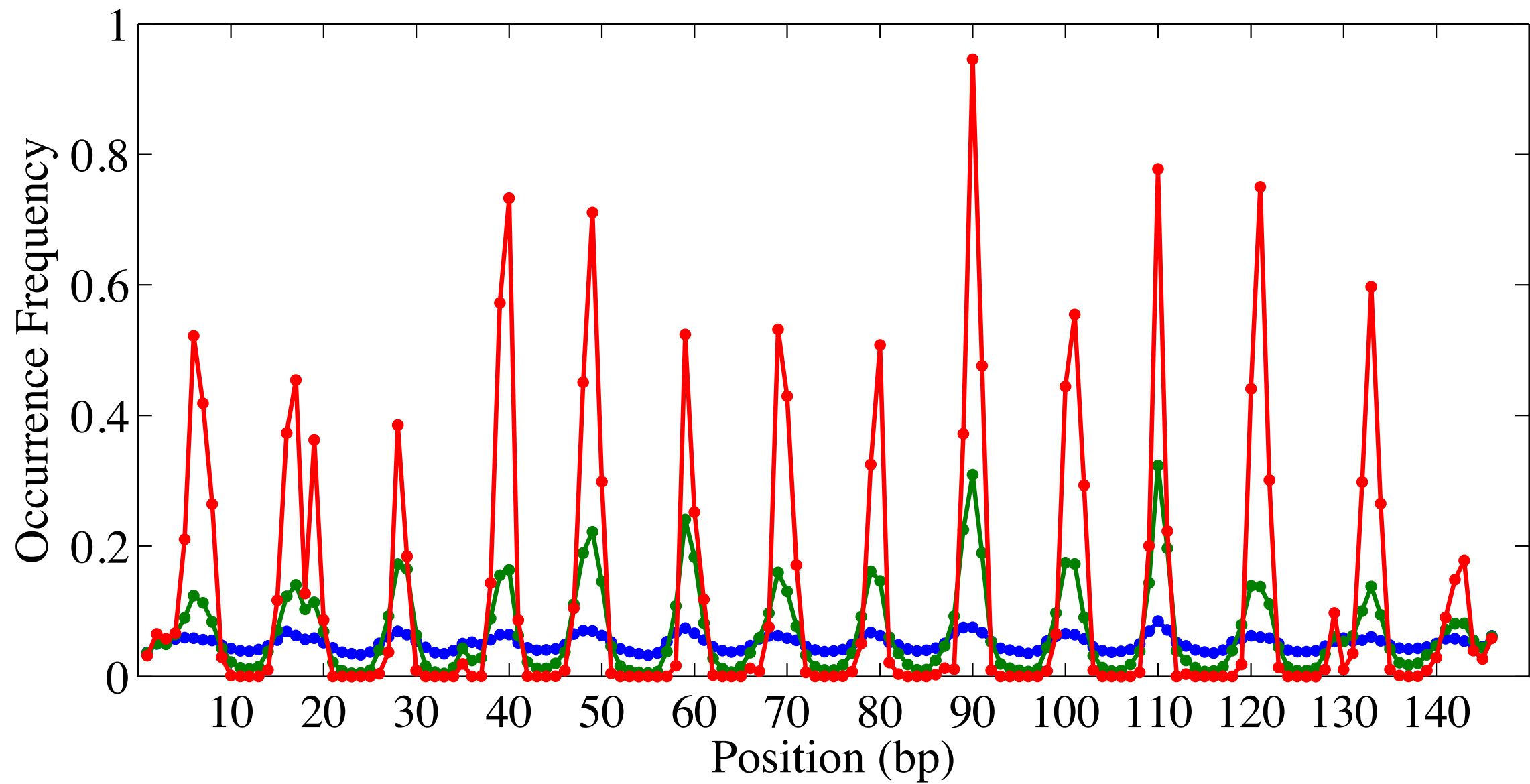

Supplement: S5 Fig — Probability distribution of the AA step, obtained by the MMC for three different temperatures: T = 600 K (blue), T = 100 K (green) and T = 21 K (red). (PDF) [file pone.0156905.s007.pdf]

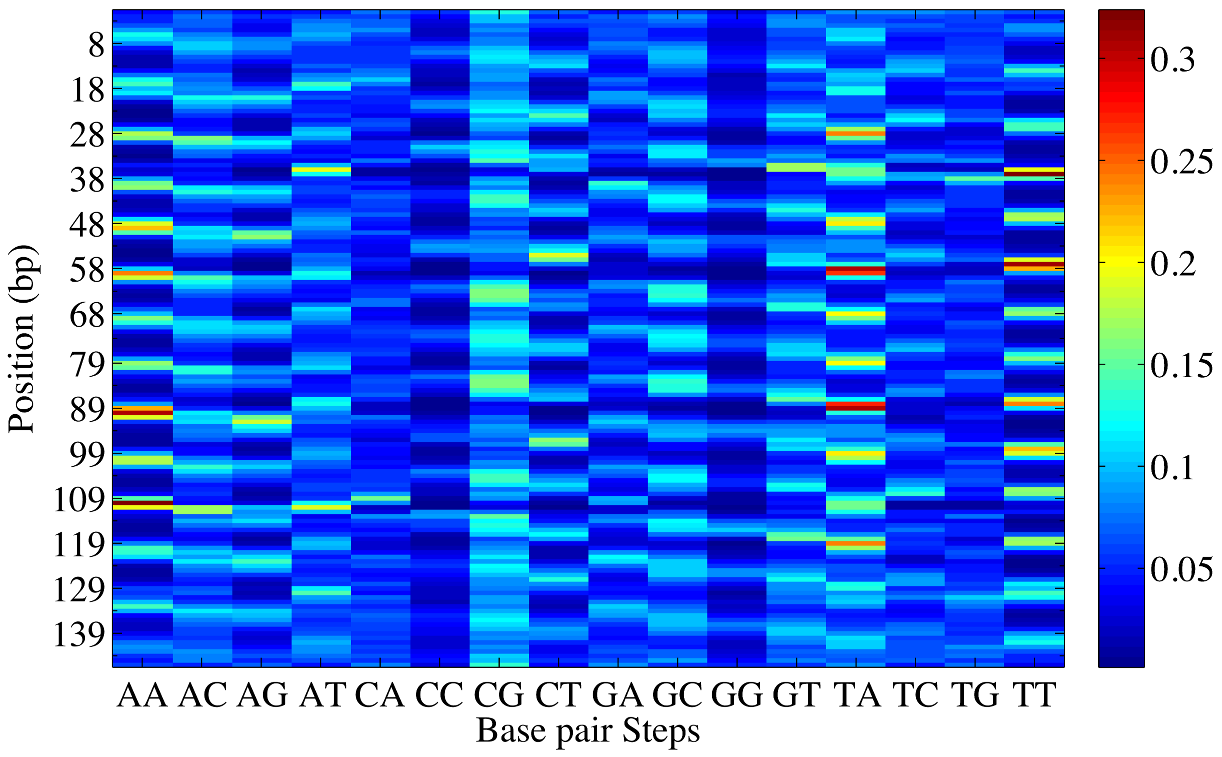

Supplement: S6 Fig — The distributions are obtained in a Mutation Monte Carlo simulation at temperature 100 K. (TIFF) [file pone.0156905.s008.tiff]

Frequency

**A**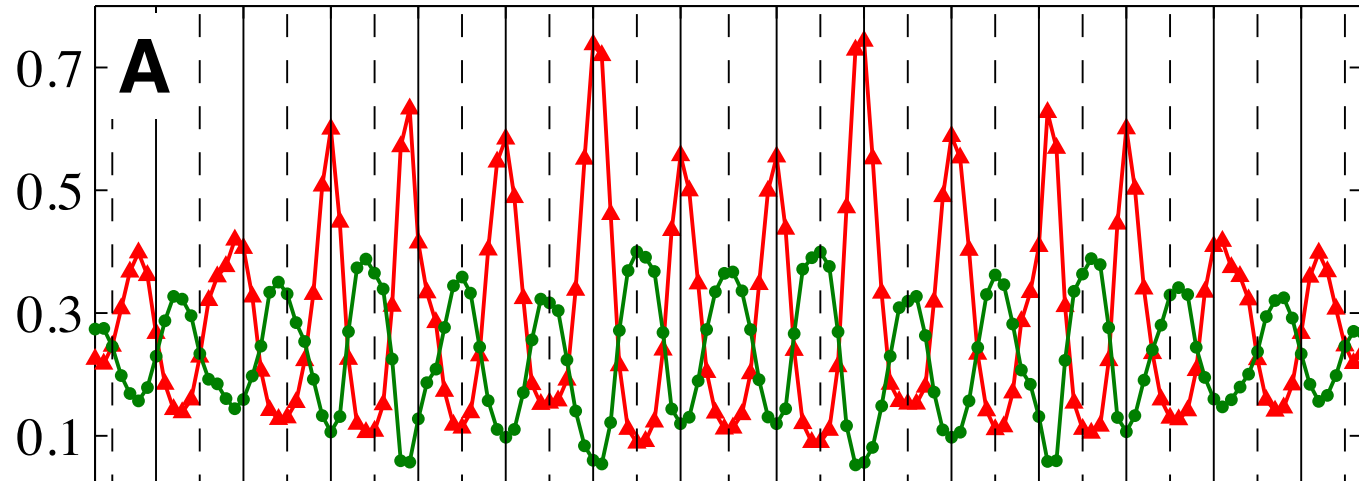

Frequency

**B**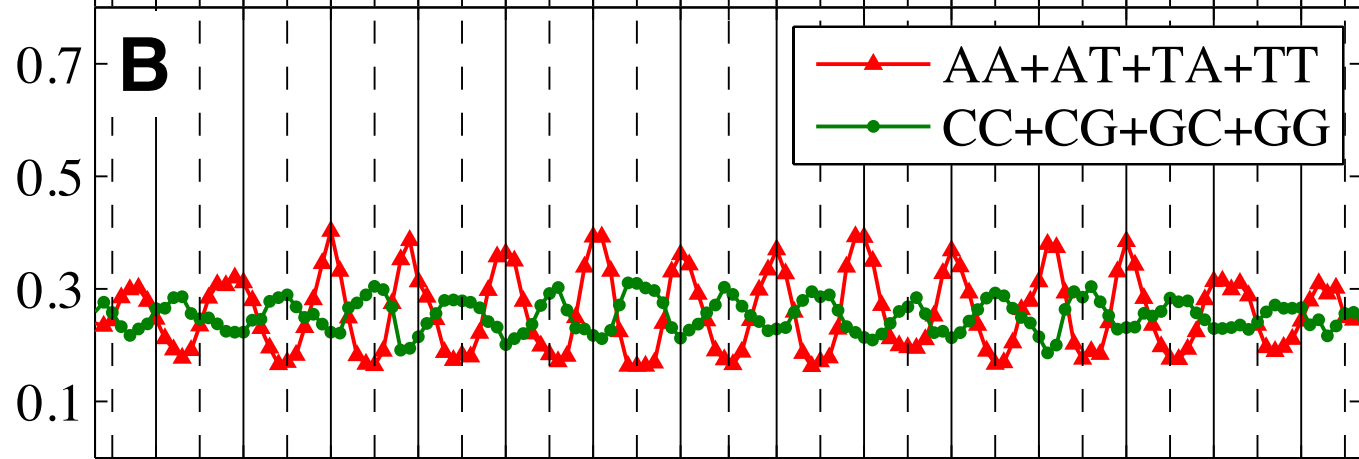

—▲— AA+AT+TA+TT  
—●— CC+CG+GC+GG

Position (bp)

Supplement: S7 Fig — (A) Fraction of dinucleotides AA/AT/TA/TT and separately CC/CG/GC/GG at each position along the nucleosome model found in 10 million high affinity sequences produced by MMC at 100 K. The model recovers the basic nucleosome positioning code. (B) Same as (A) but on top of 1200 randomly generated coding sequences (produced by sMMC). The same periodic signals are found albeit with a smaller amplitude. (PDF) [file pone.0156905.s009.pdf]

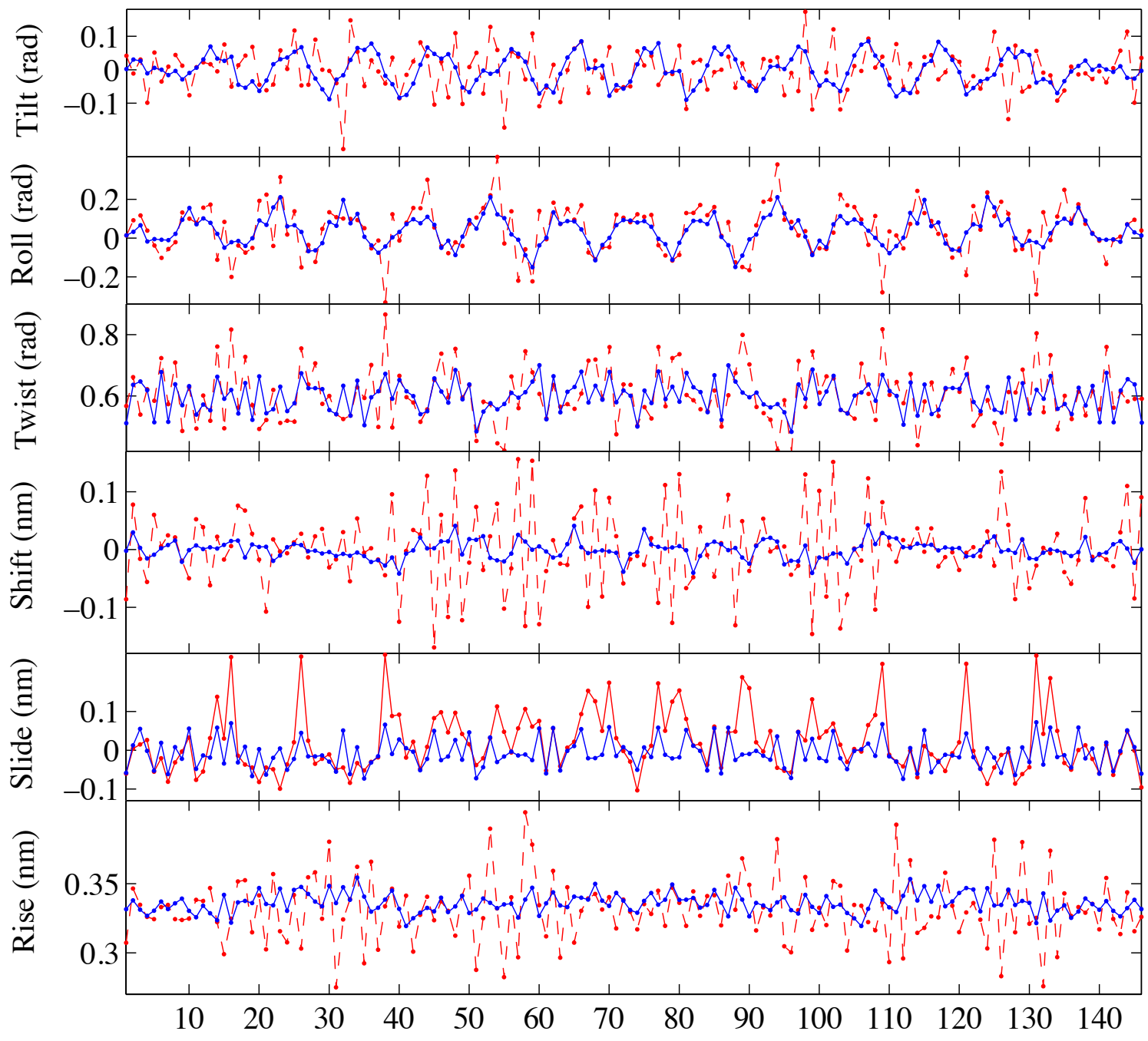

Supplement: S8 Fig — The averaged degrees of freedom for NCP147 DNA sequence as obtained in the model (solid curves, blue), in comparison with the crystal structure (dashed curves, red) [16]. (PDF) [file pone.0156905.s010.pdf]

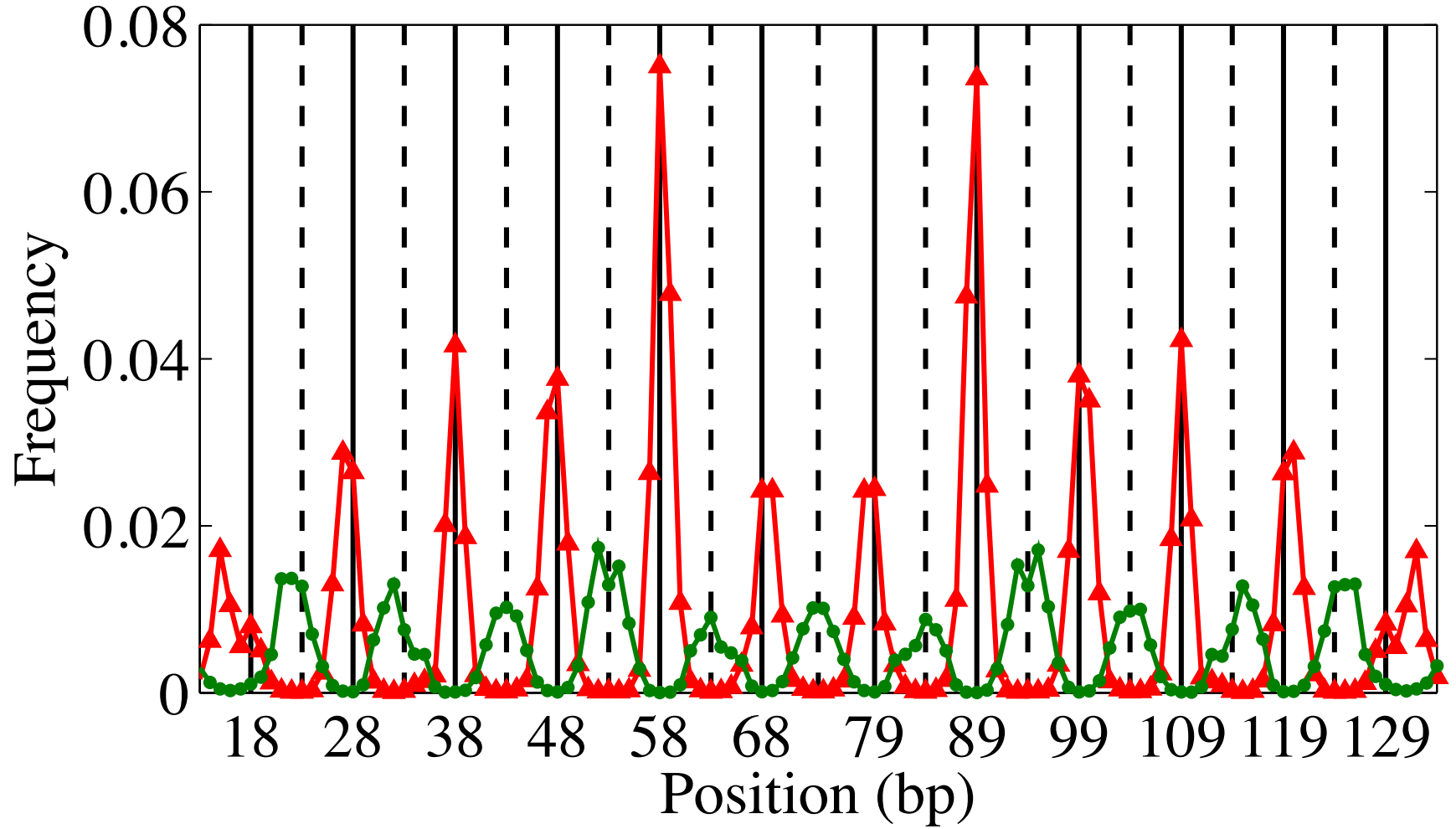

Supplement: S9 Fig — The solid and dashed vertical lines indicate minor and major groove bending sites respectively. (PDF) [file pone.0156905.s011.pdf]

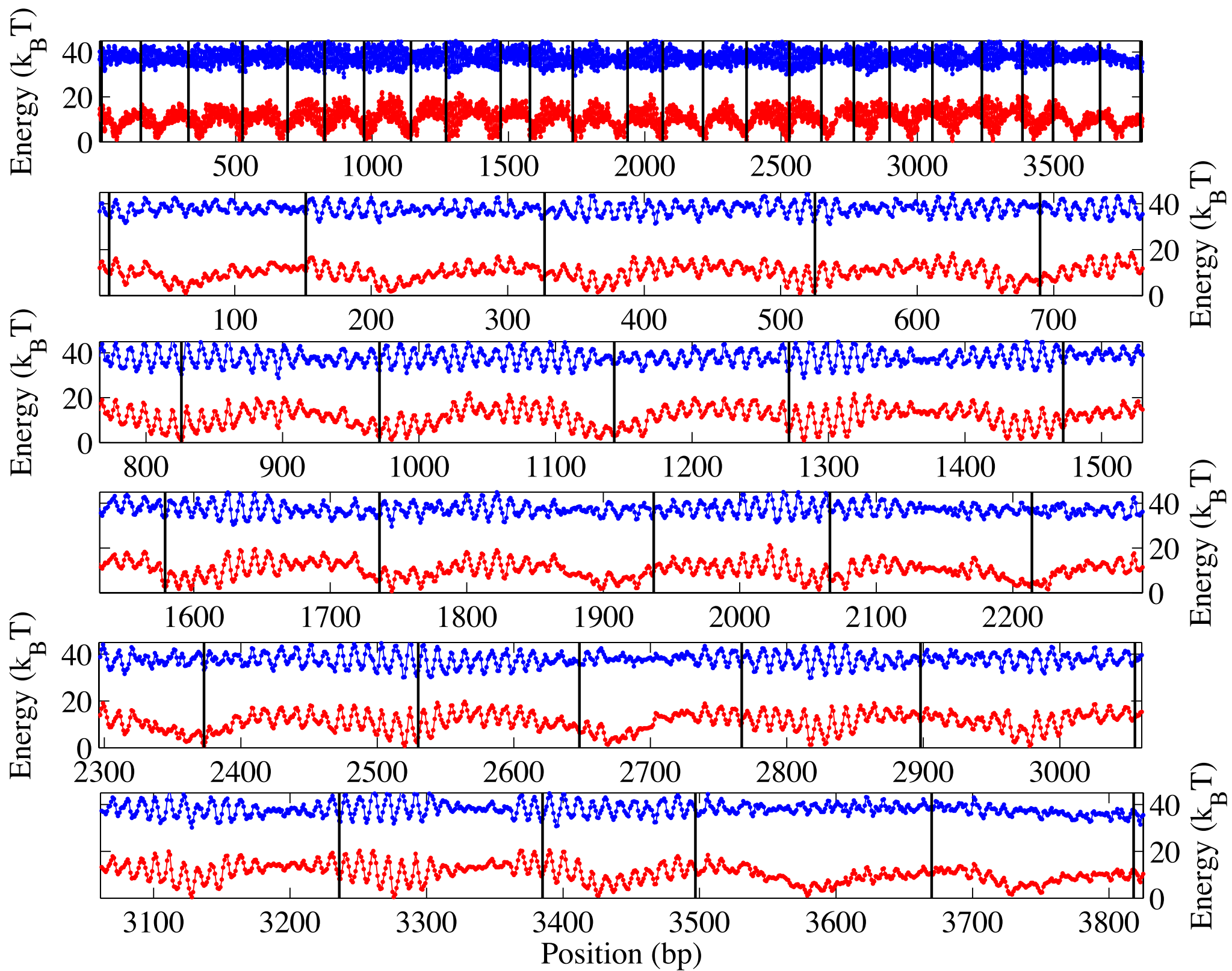

Supplement: S10 Fig — The effective energy landscape with μ = 80kT (red curves), the elastic energy landscape (blue curves) and the experimentally mapped nucleosomes [25] (vertical black lines) along the YAL002W yeast gene. The elastic energy is shifted down by 30kT for clarity. The top panel shows the landscapes over the entire gene. Each of the remaining panels zooms into a 765 bp long portion of the gene. All of the experimentally mapped nucleosome positions fall into local minima. In addition, the corresponding minima are quite deep in the central region of the gene. (PDF) [file pone.0156905.s012.pdf]

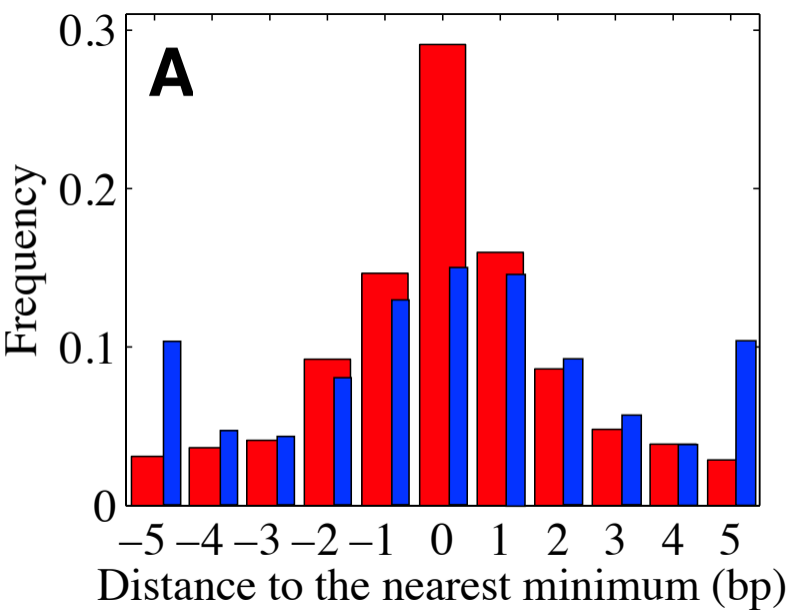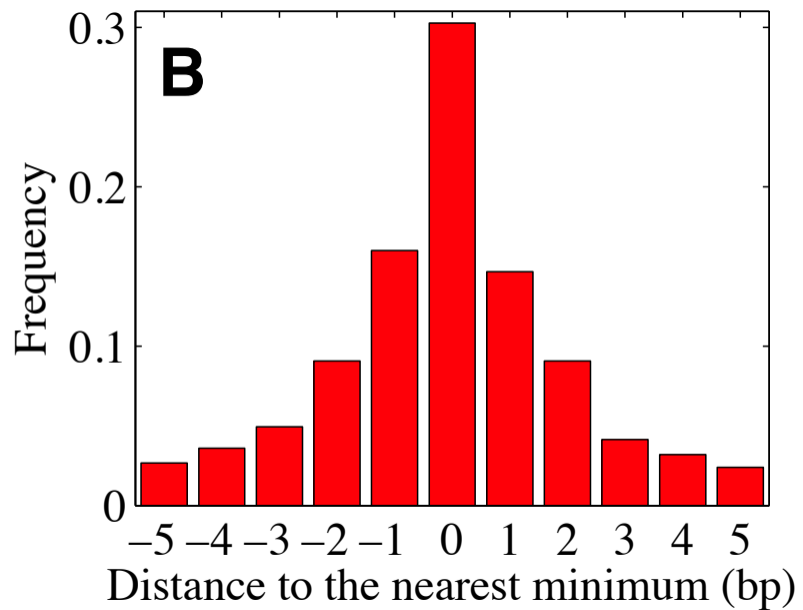

Supplement: S11 Fig — (A) The histogram of the distances between 1293 experimentally mapped nucleosomes [25] on yeast chromosome I and the nearest local minima in the theoretical energy landscape (red rectangles). As a comparison we show also the prediction from a probabilistic model trained on in vitro data (blue rectangles) [27]. (B) The distance histogram as defined in (A) for 769 nucleosomes on yeast chromosome I which are located on the genes. The two histograms are quite similar. In both cases, 60 percent of the experimental nucleosome positions lie within the range of one bp around a local minimum in the theoretical energy landscape. (PDF) [file pone.0156905.s013.pdf]

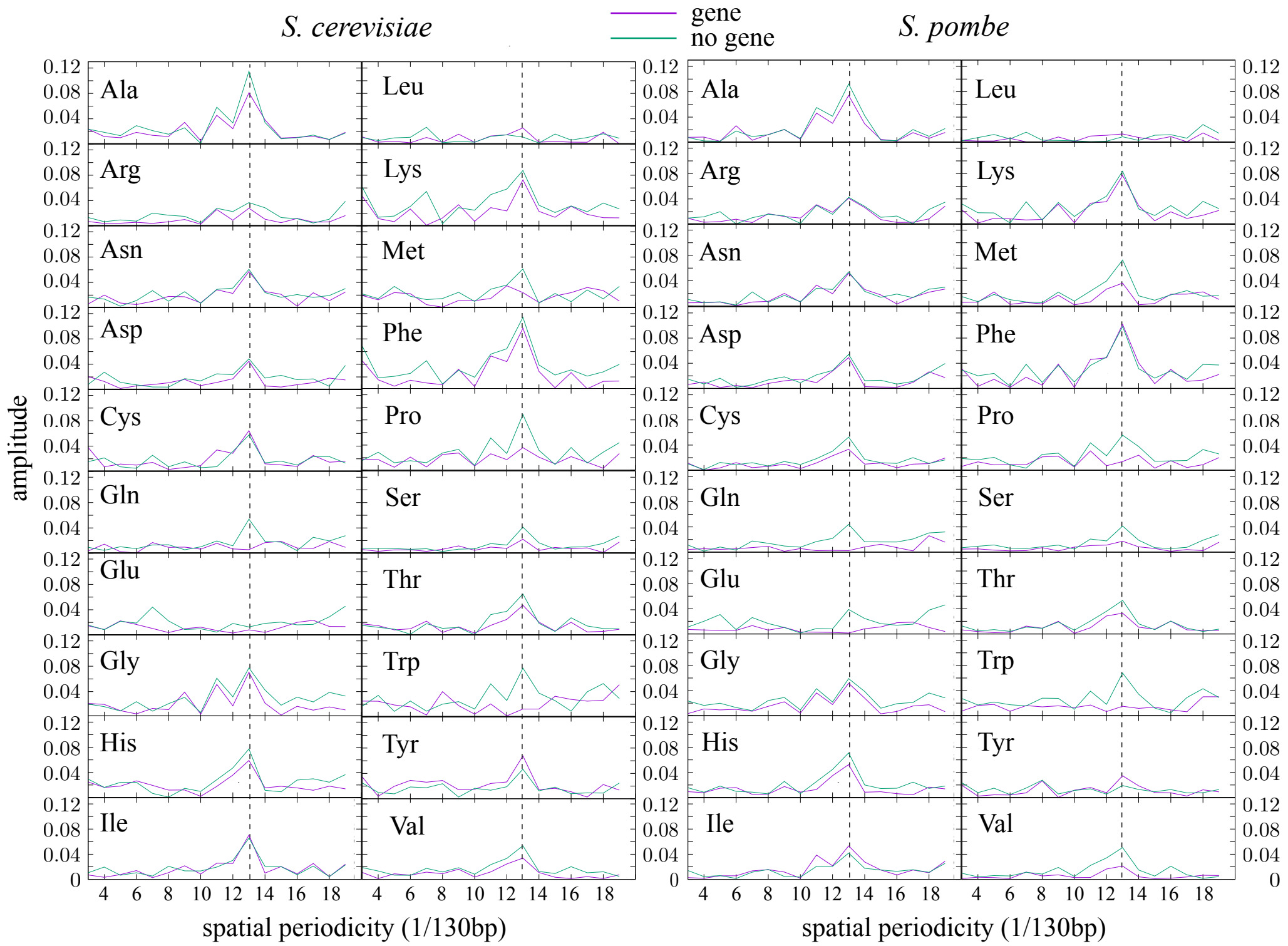

Supplement: S12 Fig — Normalized Fourier amplitudes of the distribution of the synonymous codons for all 20 amino acids along nucleosomes on top of genes (purple curve) and of the distribution of the corresponding trinucleotides along nucleosomes outside genes (blue curve) for S. cerevisiae (left) and S. pombe (right). The peaks at spatial periodicity 13 corresponds to a 10 bp periodic signal. In most cases the height of this peak is larger for the non-coding case, evidence for multiplexing of genetic and mechanical information. (PDF) [file pone.0156905.s014.pdf]
